# High Step-Up Ratio DC-AC Converter Using Fourth-Order LCLC Resonant Circuit for Ultrasonic Fingerprint-Sensor Drivers

**DOI:** 10.3390/mi14020393

**Published:** 2023-02-04

**Authors:** Wei Liu, Yunlai Shi, Zhijun Sun, Li Zhang, Qian Zhang

**Affiliations:** State Key Laboratory of Mechanics and Control of Mechanical Structures, Nanjing University of Aeronautics and Astronautics, Nanjing 210016, China

**Keywords:** ultrasonic fingerprint sensor, fourth LCLC resonant converter, high step-up ratio

## Abstract

Ultrasonic fingerprint sensors are becoming more widely used in thick or flexible displays. In order to better identify fingerprint information, ultrasonic sensors need to generate more ultrasonic energy, which can be transmitted to the display surface through media with higher acoustic impedance. In this paper, a DC-AC converter with a high lift ratio was proposed to enhance the transmission energy of the ultrasonic fingerprint sensor, thus helping to improve the identification. The converter comprises a full-bridge inverter and two LC resonant circuits. The introduction of an additional LC resonant circuit into the traditional Class-D LC resonant converter effectively increases the boost ratio of the proposed converter from 5 to 22. When used as a part of the ultrasonic fingerprint sensing system, the proposed converter can amplify the 20-V low-voltage DC required to drive the piezoelectric organic film to 376 V high-voltage AC. The voltage of the wave received from this new driver is equal to 970 mV, which greatly exceeds the 376 mV achieved by using the Class-D converter alone. In this paper, the topology proposed by the ultrasonic fingerprint sensor converter driver was experimentally verified, which greatly improved the boost ratio and can be considered suitable for wider applications.

## 1. Introduction

**Figure 1:**
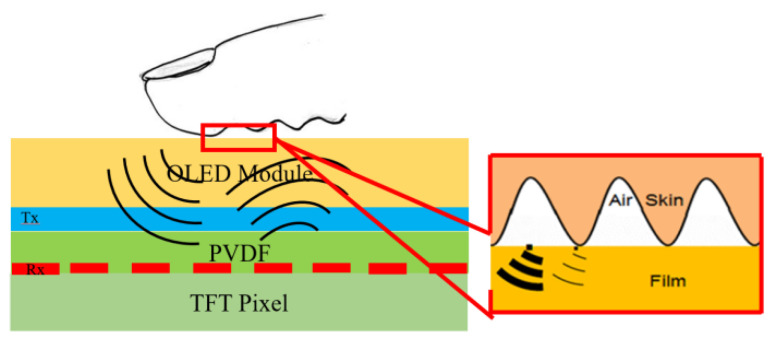
Ultrasonic fingerprint-sensor module.

Ultrasonic fingerprint-identification technology [1,2,3,4,5,6,7] has been employed to realize under-screen fingerprint identification in intelligent devices such as mobile phones and laptops. To enhance the security of fingerprint identification, large-area ultrasonic fingerprint sensors capable of simultaneously detecting multiple fingers have been proposed [4,5,6]. As illustrated in Figure 1, a typical ultrasonic fingerprint sensor installed under an organic light-emitting diode (OLED) display module comprises a thin-film transistor backplane, transmitter (Tx) electrode (upper electrode), receiver (Rx) electrode (lower electrode), and poly(vinylidene fluoride) (PVDF) piezoelectric film [7]. Being a piezoelectric material, PVDF generates ultrasonic waves when driven by high-frequency voltage AC power [8]. Ultrasonic waves pass through intermediate media and reach the finger surface. Serrations on the finger surface result in the existence of two media types—air and skin—between the finger and fingerprint sensor (Figure 1). Due to differences in acoustic impedances between the air and skin, waves reflecting from air and skin travel back to the same ultrasonic transducer, thereby causing a voltage difference across the PVDF film.

Typically, the Tx electrode is connected to a high-voltage signal, and the Rx electrode is connected to the ground. However, only low-voltage DC power is available in most mobile systems; therefore, a DC/AC converter must be used to drive ultrasonic fingerprint sensors. Due to the limited space in mobile devices, high-power and large-volume devices such as transformers cannot be used. PVDF also generates AC voltage signals on the Rx electrode when it receives echo ultrasound waves [9]. To achieve a high Rx voltage difference across the ultrasonic transducer, it is usually driven at high-voltage AC power. However, most mobile systems can only supply low-voltage DC power; therefore, a high-frequency DC-AC converter must realize a high step-up ratio to generate a high-voltage AC signal to drive ultrasonic fingerprint sensors.

**Figure 2:**
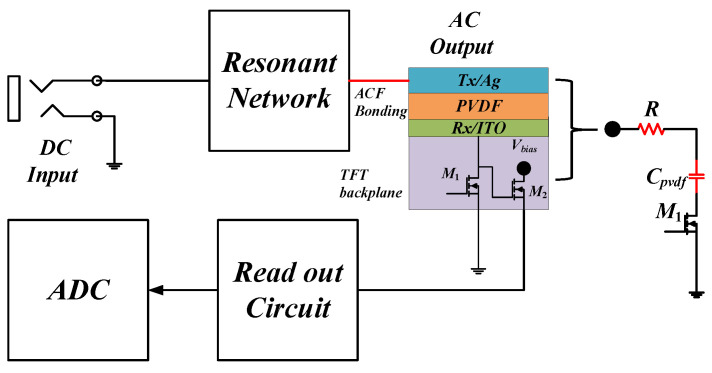
Tx and Rx circuit with a pixel.

An ultrasonic fingerprint sensor comprises thousands of pixels, and each pixel is connected to the pixel circuit, as depicted in Figure 2. A Ag Tx electrode sputtered on the PVDF film connects to the resonant network through an anisotropic conductive film (ACF) via a bonding process. The contact resistance R of ACF approximately equals 5 Ω. The Rx electrode is made of indium tin oxide (ITO) manufactured via the TFT process. Each switching cycle comprises two sub-modes—Tx and Rx. In the Rx mode, the Tx electrode is switched to ground, and the Rx electrode is connected to the thin-film transistor M2 and peak detect diode D. The voltage difference on the PVDF film generated by different acoustic impedances between air and skin causes a drain–source current difference in M2. Thus, the read-out current and ADC facilitate the accumulation of finger-image data via the detection of the drain–source current difference corresponding to M2. In the Tx mode, the DC-AC converter generates a high-voltage AC signal not added directly to the Tx and Rx electrodes; instead, it is passed through a thin-film transistor. Meanwhile, M1 must be connected to ground in light of the low normal operating voltage of TFT [3]. Otherwise, TFT would burn out due to the application of excess voltage across it. In the Tx mode, the equivalent circuit of a pixel comprises a bonding-resistor R connected in series with a PVDF capacitor CPVDF and TFT M1. In summary, the DC-AC converter must be characterized by high-frequency operation, high step-up ratio, one end of the CPVDF grounded, and small form-factor.

High-power inverters have already been used to drive ultrasonic transducers in recent studies [10,11]. However, these inverters usually consist of large-volume power devices and cannot be used in mobile phones. In our previous work [12,13], a Class-D inverter was proposed for inductor heating applications. This type of inverter is a classical DC/AC converter that can also be used to drive piezoelectric ultrasonic transducers [14,15,16,17], the output voltage of which is, however, insufficient due to its low step-up ratio. This induces a weak Rx signal on the PVDF ultrasonic sensor. To increase the voltage of the Rx signal, the step-up ratio of this DC/AC converter must be improved. The traditional Class-D LC resonant converter possesses only one LC resonant loop, the quality factor of which determines the step-up ratio of this converter. In this paper, a converter with two LC resonant circuits was proposed, the step-up ratio of which was approximately the product of the quality factors of two resonant loops. The step-up ratio of the proposed converter was found to be greater than that of traditional Class-D LC converters when the quality factor of the second resonant circuit exceeded one. This paper describes a high step-up ratio DC/AC converter using a fourth-order LCLC resonant circuit by adding another set of LC resonant circuits. The step-up ratio can be increased by approximately three times compared with that of the conventional Class-D LC resonant converter, which is expected to be sufficiently powerful to drive PVDF ultrasonic transducers. 

The remainder of this paper is organized as follows. Section 2 presents the circuit configuration and operating principle of the proposed fourth-order LCLC resonant converter. Section 3 presents the equivalent circuit analysis of the proposed converter. Section 4 presents the experimental results based on the preliminary application of the proposed converter to a prototype ultrasonic fingerprint sensor. Section 5 concludes the paper.

## 2. Fourth-Order LCLC Resonant Converter

### 2.1. Circuit Description

**Figure 3:**
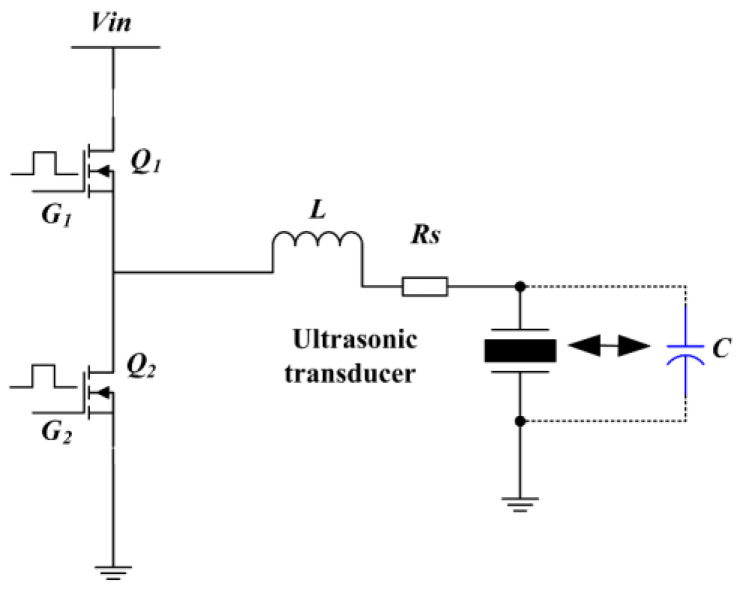
Schematic of a traditional Class-D converter.

Figure 3 depicts the schematic of a Class-D inverter proposed in extant studies [13,14,15,16]. The output power stage comprises a signal-phase voltage-source half-bridge inverter comprising two MOSFET modules—Q_1_ and Q_2_. The output from the inverter is connected to a LC resonant circuit comprising an ACF contact resistor *R*_s_, resonant inductor *L*, and PVDF ultrasonic transducer that can be modeled using a capacitor C. The parasitic resistance of inductor *L* is much lower compared to the ACF contact resistance. Therefore, in the proposed study, the same was not considered during the theoretical analysis. The piezoelectric thin film transducer is a key component in the ultrasonic fingerprint sensor, and its electrical characteristics can be modeled by a parallel plate capacitor. For a 150 mm^2^ × 30 um PVDF thin film ultrasonic transducer, the equivalent capacitance was calculated as 500 pF. According to [12,13], the resonant frequency of the LC circuits, step-up ratio of the Class-D converter, current of the inductor, and voltage of the capacitor can be expressed as follows:

(1) $$\left\{\begin{array}{l} f=1/(2\pi \sqrt{LC})\\ D_{c}=V_{c}/V_{in}=1/(2\pi fCR) \end{array}\right.$$

**Figure 4:**
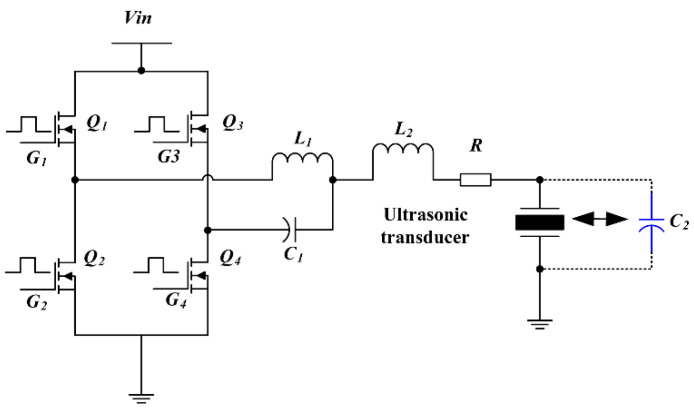
Schematic of the proposed converter.

Figure 4 shows the simplified schematic of the fourth-order LCLC resonant converter circuit. The output power stage comprises a signal-phase voltage-source full-bridge inverter with four MOSFET modules Q_1_, Q_2_, Q_3_, and Q_4_. G_1_, G_2_, G_3_, and G_4_ represent the driving signals of the MOSFETs. The output of the inverter is connected to the fourth-order LCLC resonant circuit, which consists of an ACF contact resistor *R*, two resonant inductors *L*_1_ and *L*_2_, one resonant capacitor *C*_1_, and one ultrasonic transducer modeled by a capacitor *C*_2_. 

### 2.2. Resonant Frequency

**Figure 5:**
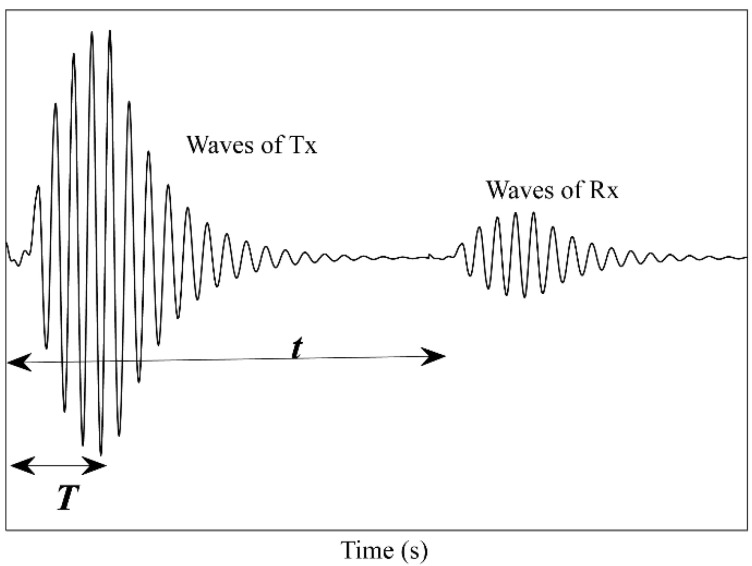
Transmitting and receiving waveforms.

Figure 1 shows the schematic of an ultrasonic fingerprint sensor module. The ultrasound waves transmitted by the PVDF film pass through the OLED module to reach the finger and are reflected by the surface of the finger. The reflected waves pass through the OLED module again, and reach the piezoelectric PVDF film, which converts the received ultrasound waves into electrical voltage signals. The transmitting and receiving waveforms are shown in Figure 5. The receiving waves do not overlap with the transmitting waves if the transmission time T is less than the transfer time *t*. 


(2) $$\left\{\begin{array}{l} T<t\\ T=N/f\\ t=2s/v \end{array}\right.$$

In Equation (2), the thickness of the OLED module *s* is approximately 1.5 mm. The transmission speed of ultrasound waves in the OLED module *v* is approximately 5000 m/s, considering that the majority of the OLED module is glass. Therefore, the switching frequency *f* must exceed *N*/0.6 MHz, which is approximately 12 MHz in this study.

### 2.3. Circuit State Analysis

**Figure 6:**
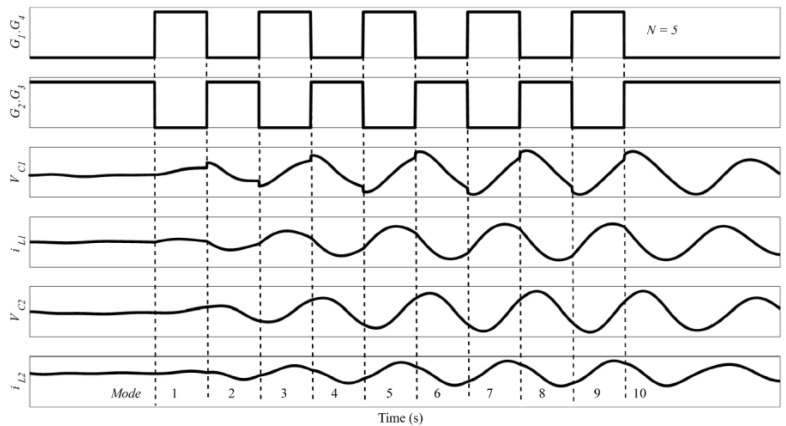
Relevant voltage and current operating waveforms.

**Figure 7:**
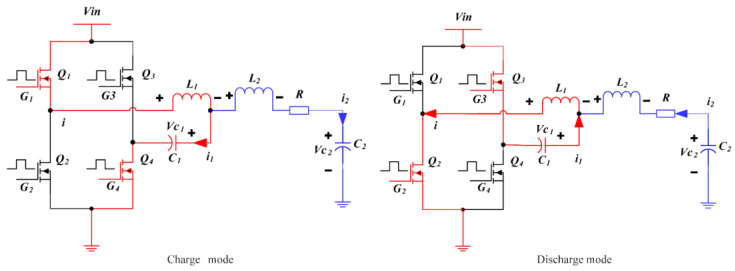
Mode transition in one switching cycle.

**Figure 8:**
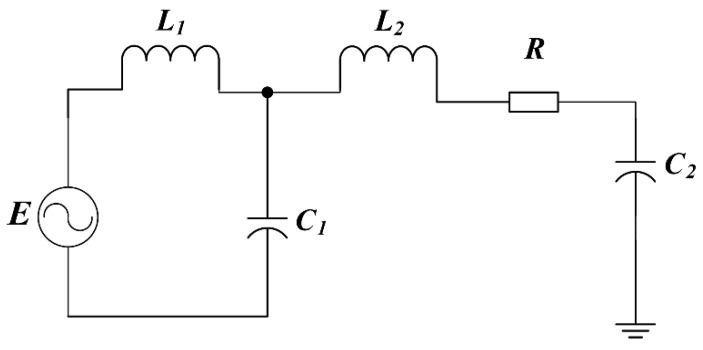
Resonant network of the converter in Figure 7.

The operating waveforms of the proposed fourth-order LCLC resonant converter are shown in Figure 6. The switch-mode transition and the resonant current pathway of the fourth-order LCLC resonant converter are depicted in Figure 7. In this converter, there are two resonant loops, which are shown in Figure 8, where one part of the inductor *L*_1_ flows through the capacitor *C*_2_ and the other flows through *L*_2_, *R*, and *C*_2_. 

The resonant circuit shown in Figure 8 can be described by the following parameters. The output voltage of *C*_2_ is given by

(3) $$\left\{\begin{array}{l} V_{L1}+V_{c1}=E\\ V_{L1}+V_{L2}+V_{R}+V_{c2}=E \end{array}\right.$$

where $\left\{\begin{array}{l} V_{L1}=i_{1}j\omega L_{1},V_{c1}=i_{1}/(j\omega C_{1}),V_{c2}=i_{2}/(j\omega C_{2})\\ V_{L2}=i_{1}j\omega L_{2},V_{R}=i_{2}\times R,i=i_{1}+i_{2} \end{array}\right.$ and *E* is the amplitude of the input power. Therefore, the open loop transfer function of *V_c_*_2_ and *E* can be represented as

(4) $$\left\{\begin{array}{l} H(j\omega )=V_{c2}(j\omega )/E(j\omega )=1/(a+bj)\\ a=[L_{1}C_{1}L_{2}C_{2}\omega^{4}-(L_{1}C_{1}+L_{1}C_{2}+L_{2}C_{2}\;)\omega^{2}+1]\\ b=(RC_{2}\omega -L_{1}C_{1}RC_{2}\omega^{3}) \end{array}\right.$$

The peak-to-peak voltage of *V_c_*_2_ and the step-up ratio of the full bridge fourth-order LCLC resonant converter are

(5) $$V_{C2}=2E\times \left|H(j\omega )\right|,D_{c2}=2/\left|H(j\omega )\right|=2/\sqrt{a^{2}+b^{2}}$$

If the number of pulses is *N*, a transmitter cycle operation can be divided into 2*N* sub-modes, as shown in Figure 6. There are two sub-modes in one switching cycle: charge and discharge. The operating principles of the charge and discharge modes are as follows. 

(1) Mode 1 [0, T/2]: The circuit mode of the fourth-order LCLC resonant converter enters the charge mode. Q_1_ and Q_4_ are conducted, and the capacitors *C*_1_ and *C*_2_ start charging. According to the KVL law, the initial values of *i*_1_, *i*_2_, *V_c_*_1_, and *V_c_*_2_ are zero, and the state equations of this mode are expressed as follows:

(6) $$\left\{\begin{array}{l} \frac{L_{1}d(C_{1}\frac{dV_{c1}}{dt}+C_{2}\frac{dV_{c2}}{dt})}{dt}+V_{c1}=E\\ \frac{L_{1}d(C_{1}\frac{dV_{c1}}{dt}+C_{2}\frac{dV_{c2}}{dt})}{dt}+\frac{L_{2}C_{2}d^{2}V_{c2}}{dt^{2}}+\frac{RC_{2}dV_{c2}}{dt}+V_{c2}=E\\ V_{c1}(1,0)=0\\ V_{c2}(1,0)=0 \end{array}\right.$$

(2) Mode 2 [T/2, T]: At T/2, Q_1_ and Q_4_ are turned off, and Q_2_ and Q_3_ are turned on. The circuit mode of the fourth-order LCLC resonant converter switches to the discharge mode. The discharge mode initiates at the end of the charge mode. Therefore, the state equations of this mode are expressed as follows:

(7) $$\left\{\begin{array}{l} \frac{-L_{1}d(C_{1}\frac{dV_{c1}}{dt}+C_{2}\frac{dV_{c2}}{dt})}{dt}=V_{c1}+E\\ \frac{-L_{1}d(C_{1}\frac{dV_{c1}}{dt}+C_{2}\frac{dV_{c2}}{dt})}{dt}-\frac{L_{2}C_{2}d^{2}V_{c2}}{dt^{2}}-\frac{C_{2}RdV_{c2}}{dt}=V_{c2}\\ V_{c1}(2,0)=V_{c1}(1,T/2)\\ V_{c2}(2,0)=V_{c2}(1,T/2) \end{array}\right.$$

In one switching cycle, the converter transitions from Mode 1 to Mode 2, and then back to Mode 1 in the next switching cycle. If the system transmits *N* pulses, the converter repeats this action *N* times. Thus, Modes 3, 5, 7, and 9 have the same state equations as Mode 1, and Modes 4, 6, 8, and 10 have the same state equations as Mode 2. However, the initial value of each state differs because the end value of the previous state will be different. The state Equations (6) and (7) have the same characteristic equation:

(8) $$\begin{array}{l} s^{4}+\frac{R}{L_{2}\;}s^{3}+\frac{\left(L_{1}C_{1}+L_{1}C_{2}+L_{2}C_{2}\;\right)}{L_{1}C_{1}L_{2}C_{2}\;}s^{2}\\ +\frac{R}{L_{1}C_{1}L_{2}\;}s+\frac{1}{L_{1}C_{1}L_{2}C_{2}\;}=0 \end{array}$$

The characteristic roots of Equation (8) are *a*_1_ ± *b*_1_*i* and *a*_2_ ± *b*_2_*i*. The voltage and current waveforms of Mode *n* in the time domain can be obtained by solving Equations (6)–(8). 

When *n* is an odd number, *V_c_*_2_ is

(9) $$\begin{array}{l} V_{c2}(n,t)=e^{a_{1}t}[A\times \sin(a_{1}t)+B\times \cos(b_{1}t)]\\ +e^{a_{2}t}[C\times \sin(a_{2}t)+D\times \cos(b_{2}t)]+E \end{array}$$

When *n* is an even number, *V_c_*_2_ is

(10) $$\begin{array}{l} V_{c2}(n,t)=e^{a_{1}t}[A\times \sin(a_{1}t)+B\times \cos(b_{1}t)]\\ +e^{a_{2}t}[C\times \sin(a_{2}t)+D\times \cos(b_{2}t)]-E \end{array}$$

The initial value of each Mode is

(11) $$\begin{array}{l} \left\{\begin{array}{l} i_{L1}(n,0)=i_{L1}(n-1,0.5T)\\ i_{L2}(n,0)=i_{L2}(n-1,0.5T)\\ V_{C1}(n,0)=V_{C1}(n-1,0.5T)\\ V_{C2}(n,0)=V_{C2}(n-1,0.5T) \end{array}\right.,(n>1)\\ \left\{\begin{array}{l} i_{L1}(n,0)=0,i_{L2}(n,0)=0\\ V_{C1}(n,0)=0,V_{C2}(n,0)=0 \end{array}\right.,(n=1) \end{array}$$

where *A*, *B*, *C*, and *D* can be obtained by solving the initial (11). *i*_1_, *i*_2_, *V_c_*_1_ can be obtained by solving Equations (6), (7), (9) and (10), which are described as follows:

(12) $$\left\{\begin{array}{l} i_{2}(n,t)=C_{2}y^{\prime };y=V_{c2}(n,t)\\ V_{c1}(n,t)=L_{2}C_{2}\;y^{\prime \prime }+RC_{2}y^{\prime }+y\\ i_{1}(n,t)=C_{1}L_{2}C_{2}\;y^{\prime \prime \prime }+RC_{1}C_{2}y^{\prime \prime }+C_{1}y^{\prime }\\ i(n,t)=C_{1}L_{2}C_{2}\;y^{\prime \prime \prime }+RC_{1}C_{2}y^{\prime \prime }+(C_{1}{+C}_{2})y^{\prime } \end{array}\right.$$

## 3. Analysis of Fourth-Order LCLC Resonant Converter

### 3.1. Analysis of Step-Up Ratio

When the switching frequency *f* = 12 MHz and load capacitor *C*_2_ = 500 pF, the step-up ratio of the Class-D converter can be calculated by substituting Equation (13) into Equation (1). 


$$D_{C}=1/\left(2\pi fCR\right)=5.3052$$

Similarly, when the inductors in the fourth-order LCLC converter *L*_1_ = 100 nH and *L*_2_ = 110 nH, the step-up ratio can be calculated by substituting Equation (13) into Equation (5).

$$D_{c2}=2/\left|H(j\omega )\right|\approx 22>D_{C}=5.3052$$

**Figure 9:**
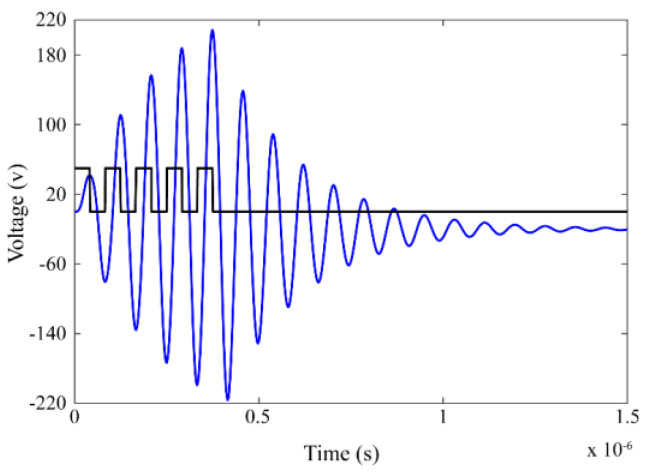
Voltage waveforms of *V_c_*_2_ for *n* = 5.

By comparing the step-up ratios of the fourth-order LCLC converter *D_c_*_2_ and the Class-D converter *D_c_*, we observed that the step-up ratio of the proposed converter was approximately four times larger than that of the Class-D inverter. According to previous studies in [12,13], the peak-to-peak voltage of the resonant capacitor cannot exceed 106 V with the resonant parameters proposed in this paper. However, in this proposed converter, the peak-to-peak voltage of the resonant capacitor was considerably greater than 106 V. Figure 9 shows the resonant voltage waveforms of *V_c_*_2_, which were obtained by solving Equations (9)–(12) in MATLAB. The peak-to-peak voltage of *V_c_*_2_ gradually increased from 0 to 420 V as the number of pulses increased from 0 to 5. This indicates that the proposed fourth-order LCLC converter is a high step-up ratio converter.

### 3.2. Analysis of Resonant Circuit Parameters

The proposed fourth-order LCLC resonant converter can be considered as two resonant circuit loops connected in series. One resonant loop comprises an inductor *L*_1_ and a capacitor *C*_1_, and the other loop comprises two inductors *L*_1_ and *L*_2_, two capacitors *C*_1_ and *C*_2_, and a serial resistor *R*. The value of *C*_2_ is determined by the device structure, which is 500 pF. The fourth-order LCLC resonant converter has two resonant frequency points, which can be expressed as follows.

(13) $$f_{1}\approx 1/(2\pi \sqrt{L_{1}C_{1}}),f_{2}\approx 1/[2\pi \sqrt{(L_{1}+L_{2})C_{2}}]$$

**Figure 10:**
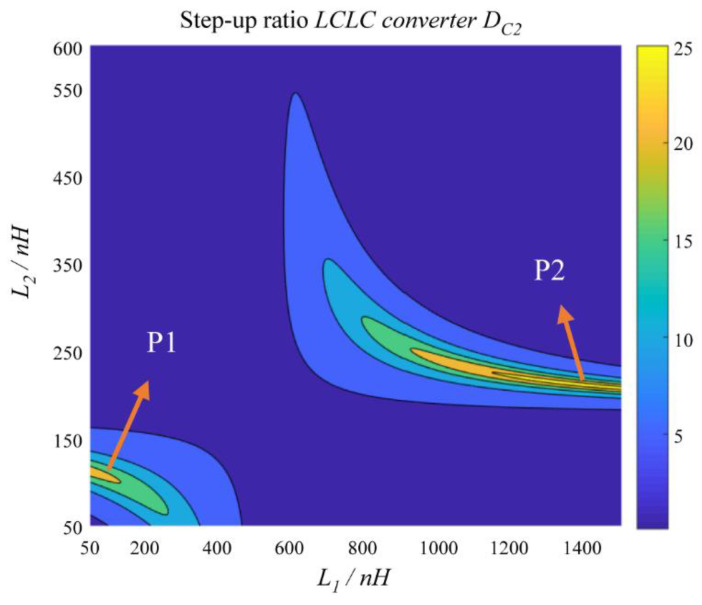
Effect of L1 and L2 on the step-up ratio *D*_c2_.

The resonant circuit analysis in our previous works [12,13] revealed that the voltage on the resonant capacitor was the maximum when the switching frequency was approximately equal to the resonant frequency. Thus, the step-up ratio *D*_c2_ has two maximum points, as shown in Figure 10. When *L*_1_ and *L*_2_ increase from 50 nH and *C*_2_ = 500 pF, *R* = 5, *f* = 12 MHz, and *C*_1_ = 980 pF, the resonant frequency *f*_2_ in Equation (13) is approximately equal to the switching frequency *f*. At this time, the step-up ratio *D*_c2_ reaches the first maximum point P_1_. As *L*_1_ and *L*_2_ increase further, the resonant frequency *f*_1_ is approximately equal to the switching frequency *f*. The step-up ratio *D*_c2_ reaches the second maximum point P_2_. 

**Figure 11:**
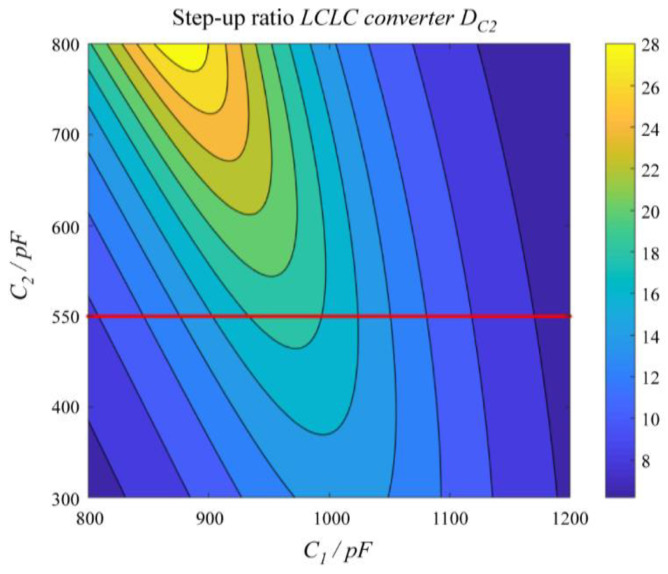
Effect of *C*_1_ and *C*_2_ on the step-up ratio *D*_c2_.

Figure 11 presents the change in the step-up ratio *D*_c2_. The step-up ratio *D*_c2_ initially increased and then decreased as the value of *C*_1_ increased from 800 pF to 1200 pF. The value of *C*_2_ was determined by the structure of the ultrasonic fingerprint sensor, which was 500 pF. The step-up ratio *D*_c2_ was the maximum when *C*_1_ was 980 pF. 

## 4. Experimental Results

**Figure 12:**
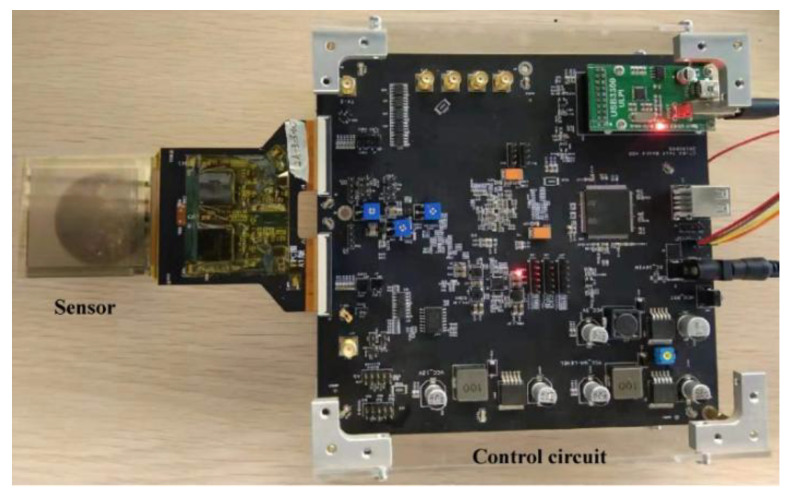
Prototype of the proposed fourth-order LCLC converter to drive an ultrasonic fingerprint sensor.

To evaluate the feasibility of the proposed fourth-order LCLC converter, circuit experiments were conducted. A full bridge LCLC resonant converter prototype was designed to drive an ultrasonic fingerprint sensor, as shown in Figure 12. The major experimental parameters are listed in Table 1. The generalized control strategy of the prototype was implemented in FPGA EP4CE75F23C8N (Altera) using Verilog HDL. 

**Figure 13:**
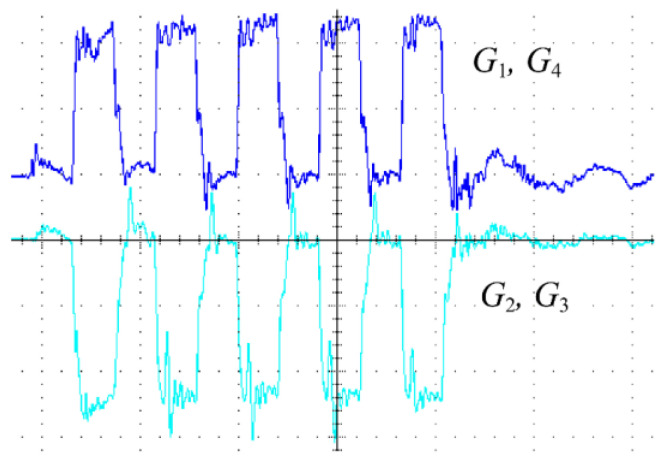
Waveforms of the MOSFET driving signals (100 nS/div 5 V/div).

The MOSFET driving signals G_1_, G_2_, G_3_, and G_4_ were generated via FPGA (see Figure 13). The blue waveform is the driving waveform of MOSFET Q_1_ and Q_4_ and the light green waveform is the driving waveform of MOSFET Q_2_ and Q_3_ in Figure 7. When G_1_ and G_4_ are high level, G_2_ and G_3_ are low level, MOSFET Q_1_ and Q_4_ are open, MOSFET Q_2_ and Q_3_ are closed, and the converter is in charge mode; when G_1_ and G_4_ are low level, G_2_ and G_3_ are high level, then MOSFET Q_1_ and Q_4_ are closed, Q_2_ and Q_3_ are open, and the converter is in discharge mode. At this time, the converter has completed a cycle of charging and discharging. In Figure 13, there are five pulse drives in total, so after five charging and discharging cycles, the MOSFETs are all closed. 

**Figure 14:**
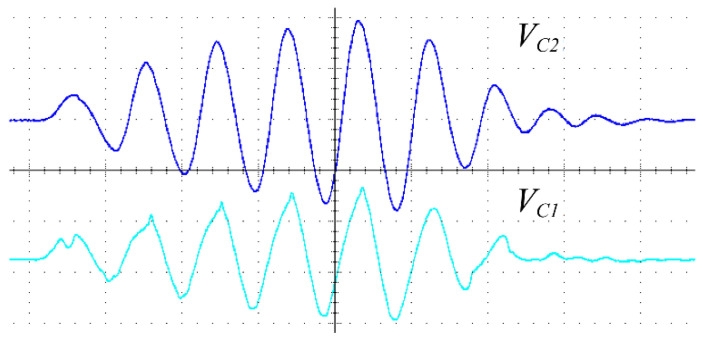
Experimental waveforms (100 nS/div 100 V/div) of resonant capacitor voltage V_C2_ (blue V_PP_ = 376 V) and V_C1_ (sky blue V_PP_ = 260 V).

The resonant voltages of the proposed converter V_C1_ and V_C2_ are depicted in Figure 14, where the blue waveform is the voltage of capacitor c1, and the green waveform is the voltage of capacitor c2. In the charging mode, V_C1_ and V_C2_ gradually increased, while in the discharge mode, V_C1_ and V_C2_ gradually decreased. After five charging and discharging cycles, V_C1_ and V_C2_ gradually increased the maximum value. With all MOSFETs closed, the resonant capacitor enters the free damping oscillation state. Under the effect of damping, V_C1_ and V_C2_ gradually decays from the highest point to 0. The experimental waveforms are identical to the theoretical waveforms, as predicted in Figure 6. The peak-to-peak voltage of V_C2_ was 376 V, which was less than the theoretical voltage 420 V. This is mainly because the parasitic resistor of MOSFET is not considered in the theoretical calculation. In the experimental circuits, these parasitic resistors can induce a reduction in the resonant voltage. Thus, the resonant voltage tested in the experimental circuits is expected to be lower than the theoretical value. 

**Figure 15:**
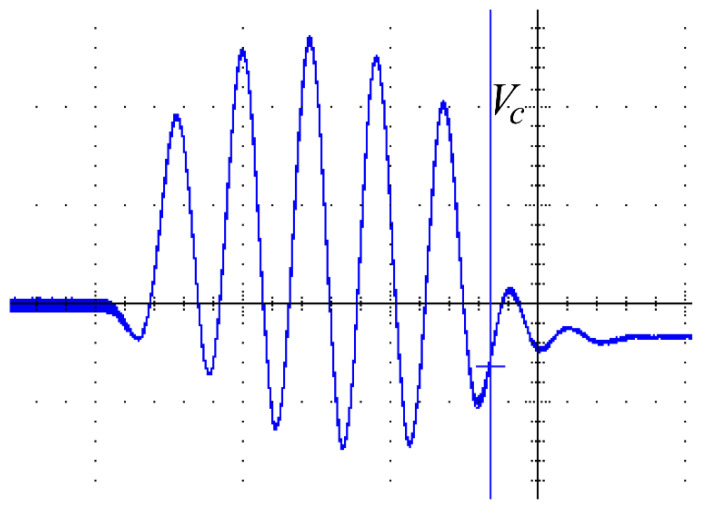
Experimental voltage waveforms of the Class-D resonant converter (200 nS/div 20 V/div). V_PP_ = 83.6 V.

The resonant voltage of the Class-D resonant converter V_c_ is shown in Figure 15. The blue waveform is the voltage output by the Class-D converter in the resonant state. The peak-to-peak voltage of V_C2_ was 86 V, which was less than the resonant voltage of the proposed converter at 376 V. This proves that the step-up ratio in the proposed fourth-order LCLC resonant converter is far greater than that in the Class-D resonant converter.

**Figure 16:**
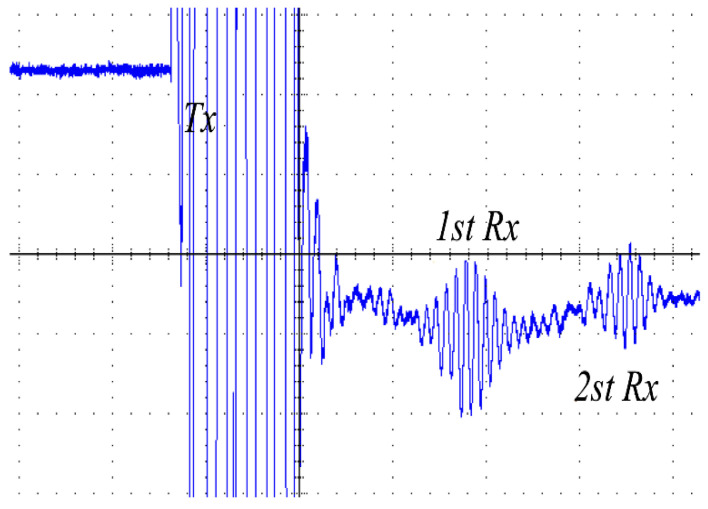
Experimental results of the receiving ultrasonic waveforms (400 nS/div 500 mV/div *Rx* V_PP_ = 970 mV).

**Figure 17:**
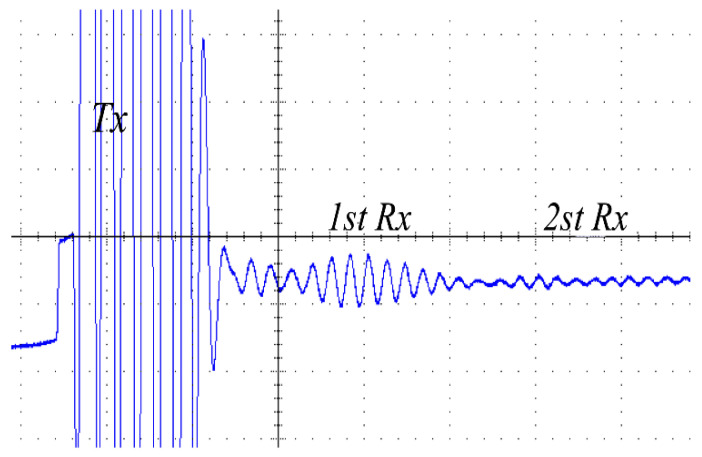
Experimental results of the receiving ultrasonic waveforms of the Class-D resonant converter (400 nS/div 500 mV/div *Rx* V_PP_ = 360 mV).

In addition, Figure 16 and Figure 17 display the receiving ultrasonic waveforms of the proposed converter and the Class-D resonant converter. The start pulse is the amplified waveform of the Vc2 voltage in Figure 14 (blue). Under the excitation of this waveform, the piezoelectric material vibrates the ultrasonic wave, so this waveform is also called Tx. The ultrasonic wave bounces back after passing through the medium, and the piezoelectric material receives the echo and converts it into an electrical signal, which is the first RX envelope waveform shown in the figure. After the echo is transmitted back through the medium again, similarly, the electrical signal generated by the piezoelectric materials can be the second echo, that is, the second Rx envelope waveform shown in the figure. The larger the echo, the better for the subsequent circuit, which can be seen from the comparison of the echoes generated under the drive of two different converters.

The peak-to-peak voltages of the waveforms in Figure 16 and Figure 17 were 980 mV and 360 mV, respectively. The magnitude of the voltage in the receiving waveforms further proves that the fourth-order LCLC resonant converter is a high step-up ratio converter. 

## 5. Conclusions

A high step-up ratio DC-AC converter based on a fourth-order LCLC circuit was proposed in this paper. The proposed converter had two resonant loops. The theoretical calculations agreed well with the experimental results, indicating that the step-up ratio of the proposed converter is considerably higher than that of the conventional Class-D converter. Under the same input voltage condition, the Class-D converter had a limited output voltage multiple in the resonant state, which was lower than the LCLC converter proposed in this paper. The higher the output voltage, the higher the vibration amplitude of piezoelectric materials, the greater the ultrasonic energy generated, and the thicker the media that can be penetrated.

In this paper, an ultrasonic fingerprint sensor prototype was constructed to verify that the proposed fourth-order LCLC resonant converter can be used in mobile phones to drive the organic piezoelectric thin film-based ultrasonic fingerprint sensors. The proposed converter enables ultrasonic fingerprint sensors to be applied to a wider range of media with higher acoustic impedances. This study also provides theoretical guidance for future applications of large-area ultrasonic sensors in different scenarios.

## Figures and Tables

**Table 1:** Parameters of the prototype.

Parameters	Symbols	Value
Input voltage	*V*_in_	20 V
Equivalent effective inductance 1	*L*_1_	100 nH
Equivalent effective inductance 2	*L*_2_	110 nH
Resonant capacitors 1	*C*_1_	980 pF
Resonant capacitors 2	*C*_2_	500 pF
ACF contact resistance	*R*	5 Ω
Switching frequency	*f*	12 MHz
Thickness of glass cover	*s*	2 mm
MOSFET switches	Q_1_, Q_1_, Q_3_, Q_4_	WSP4620

## Data Availability

Data is unavailable due to privacy.

## References

[1] Tang H.Y., Lu Y., Jiang X., Ng E.J., Tsai J.M., Horsley D.A., Boser B.E. (2016). 3-D Ultrasonic Fingerprint Sensor-on-a-Chip. IEEE J. Solid-State Circuits.

[2] Tang H.-Y., Lu Y., Assaderagh F., Daneman M., Jiang X., Lim M., Li X., Ng E., Singhal U., Tsai J.M. 11.2 3D ultrasonic fingerprint sensor-on-a-chip. Proceedings of the 2016 IEEE International Solid-State Circuits Conference.

[3] Muralt P., Ledermann N., Paborowski J., Barzegar A., Gentil S., Belgacem B., Petitgrand S., Bosseboeuf A., Setter N. (2005). Piezoelectric micromachined ultrasonic transducers based on PZT thin films. IEEE Trans. Ultrason. Ferroelect. Freq. Control.

[4] Seo W., Pi J.-E., Cho S.H., Kang S.-Y., Ahn S.-D., Hwang C.-S., Jeon H.-S., Kim J.-U., Lee M. (2018). Transparent Fingerprint Sensor System for Large Flat Panel Display. Sensors.

[5] Nelson R.B., Erhart R.A. (2014). Apparatus and Method for TFT Fingerprint Sensor. U.S. Patent.

[6] Alexander K., Kramer A. (2010). Electrostatic Discharge Protection of a Capacitive Type Fingerprint Sensing Array. U.S. Patent.

[7] Hong S.D. (2018). Embedded Active Matrix Organic Light Emitting Diode (AMOLED) Fingerprint Sensor. U.S. Patent.

[8] Yu J., Goh W.L., Arasu M.A., Je M. (2011). A 60 V, >225 °C Half-Bridge Driver for Piezoelectric Acoustic Transducer, on SOI CMOS. IEEE Trans. Circuits Syst. II Express Briefs.

[9] Horsley D.A., Rozen O., Lu Y., Shelton S., Guedes A., Przybyla R., Boser B.E. Piezoelectric micro machined ultrasonic transducers for human-machine interfaces and biometric sensing. Proceedings of the IEEE Sensors Conference.

[10] Yuan T., Dong X., Shekhani H., Li C., Maida Y., Tou T., Uchino K. (2017). Driving an inductive piezoelectric transducer with class E inverter. Sens. Actuators A Phys..

[11] Ma K.-H., Chang W.-C., Lee Y.-C. A simple CLASS-E inverter design for driving ultrasonic welding system. Proceedings of the 2009 International Conference on Power Electronics and Drive Systems (PEDS).

[12] Bi C., Lu H., Jia K., Hu J.-G., Li H. (2016). A Novel Multiple-Frequency Resonant Inverter for Induction Heating Applications. IEEE Trans. Power Electron..

[13] Hu J., Bi C., Jia K., Xiang Y. (2015). Power Control of Asymmetrical Frequency Modulation in a Full-Bridge Series Resonant Inverter. IEEE Trans. Power Electron..

[14] Agbossou K., Dion J.-L., Carignan S., Abdelkrim M., Cheriti A. (2000). Class D amplifier for a power piezoelectric load. IEEE Trans. Ultrason. Ferroelectr. Freq. Control..

[15] Giannelli P., Bulletti A., Granato M., Frattini G., Calabrese G., Capineri L. (2019). A Five-Level, 1-MHz, Class-D Ultrasonic Driver for Guided-Wave Transducer Arrays. IEEE Trans. Ultrason. Ferroelectr. Freq. Control..

[16] Dickinson T., Mathe L.K.-A., McCarthy S., Djordjev K.D., Oliveira L.D., Zhou Q. (2018). Sensor Array with Receiver Bias Electrode. U.S. Patent.

[17] Djordjev K.D., Fennell L.E., Buchan N.I., Burns D.W., Gupta S.K., Bae S. (2016). Display with Peripherally Configured Ultrasonic Biometric Sensor. U.S. Patent.

